# Effects of Warm Compresses on the Perineal Area During the Active Phase of Labor: A Quasi-Experimental Study

**DOI:** 10.7759/cureus.67825

**Published:** 2024-08-26

**Authors:** Ayah S Bqlein, Hanan Badr

**Affiliations:** 1 Obstetrics and Gynecology, Maternity and Children's Hospital, Jeddah, SAU; 2 College of Nursing, King Abdulaziz University, Jeddah, SAU

**Keywords:** active phase of labor, perineal trauma, perineal tear, reduce pain, pregnant women

## Abstract

Background

A perineal tear refers to any injury to the female genitalia during labor, which can occur spontaneously or with the use of instruments. An episiotomy, a surgical procedure, is sometimes performed to enlarge the vaginal orifice to facilitate delivery. Ineffective management of labor pain can result in negative physiological and psychosocial outcomes.

Objective

This study aims to investigate the effect of warm compresses on the perineal area during active labor.

Methods

This quasi-experimental study utilized a convenience sample, dividing 80 mothers into intervention and control groups. Participants were randomly assigned to groups after meeting the inclusion criteria and signing consent forms. The inclusion criteria encompassed pregnant women aged 18 years or older with a healthy singleton pregnancy, a cephalic presentation, and a gestational age of 37 weeks or more. The women were in the active phase of the first stage of labor (6-7 cm cervical dilation). In Part I, a data collection tool was developed to gather sociodemographic data, obstetrical history, and initial assessment information. Part II assessed perineal pain using the Visual Analogue Scale. Part III collected data on the perineal status post-delivery, and Part IV gathered information about the newborns. The research was conducted in the labor unit at East Jeddah Hospital, with data collection occurring from November 2022 to February 2023.

Results

The results of this study revealed that the mean second-stage labor pain score was 9.73 for the control group and 8.68 for the intervention group. In the third stage of labor, the mean pain score was 4.52 for the control group and 2.75 for the intervention group. During the fourth stage of labor, the mean pain score for the control group was 2.90, compared to 1.50 for the intervention group. In terms of perineal outcomes, 17 participants (42.5%) in the intervention group had an intact perineum, compared to 11 participants (27.5%) in the control group. However, there was no significant difference in the episiotomy rate between the two groups.

Conclusions

The findings of this study indicate that applying a warm compress to the perineal area during active labor can enhance perineal intactness and significantly reduce perineal pain. Future research could explore the challenges nurses encounter when applying warm compresses to the perineum during labor.

## Introduction

During labor, mothers can experience several complications, including perineal trauma, which encompasses perineal tears and episiotomy. A perineal tear is any injury to the female genitalia during labor and can occur either spontaneously or with the use of instruments [1]. Approximately 85% of vaginal births involve some degree of perineal tear [2]. According to Sarhan et al., perineal tears, a frequent complication during vaginal delivery, may result from fetal pressure [3]. Globally, 53-89% of women, particularly primigravida, experience varying degrees of perineal tears after childbirth [3].

Perineal tears can have significant long-term effects, including persistent pain, fecal and flatulent incontinence, pain during intercourse, decreased quality of life, and depression if not properly managed [4,5]. Such trauma can also lead to psychological stress and impact relationships with family.

Episiotomy, a surgical procedure to enlarge the vaginal orifice during the second stage of labor, can lead to complications such as rectovaginal fistula, breastfeeding difficulties, skin tears, unintended extension into the rectum, Bartholin’s gland damage, as well as perineal pain, edema, bleeding, hematoma, infection, and wound dehiscence. Psychological stress and chronic pelvic pain may also persist post-delivery, with rare cases of episiotomy nonhealing and endometriosis in the episiotomy scar [6].

Ineffective management of labor pain can lead to negative physiological and psychosocial outcomes. Thus, finding strategies that offer maximum pain relief and relaxation with minimal adverse effects is crucial [7].

Various techniques, such as perineal massage, warm compresses, labor positioning, perineal compression, lubrication, and analgesia, have been shown to benefit the perineal area [8]. The World Health Organization suggests that, based on available options and patient preferences, warm compresses can help prevent perineal tears and facilitate spontaneous birth during the second stage of labor.

Warm compresses applied during the second stage of labor can enhance maternal comfort by promoting vasodilation, tissue stretching, and waste product removal [3]. Studies indicate that warm compresses are associated with a higher percentage of intact perineum, reduced perineal trauma requiring suturing, and fewer episiotomies [9].

Modoor et al. [9] conducted a randomized study in which warm compresses were combined with standard hospital care for the intervention group. Their results revealed that 52% (N = 26) of the intervention group reported moderate pain, compared to only 34% (N = 17) in the control group. Additionally, 22% (N = 11) of participants in the intervention group had an intact perineum, whereas only 10% (N = 5) of the control group had an intact perineum. Furthermore, the control group exhibited a higher incidence of episiotomy (24%, N = 12) compared to the intervention group (18%, N = 9). Similarly, Akbarzadeh et al. [10] found that heat compresses are a notable intervention for shortening the second stage of labor.

Farahmand et al. [11] conducted a randomized experiment in Iran that found 25.7% (N = 19) of the intervention group, who received perineal warm compression, reported pain scores under 4. In contrast, 0.0% (N = 0) of the control group had pain scores under 4. For pain scores over 8, 14.9% (N = 11) of the intervention group experienced such levels, compared to 58.7% (N = 44) in the control group. Gaheen and Abo-Hatab [12] performed a comparative experimental research design and found that the use of warm compresses, hands-on approaches, and perineal lubricated massage during the second stage of labor improved perineal outcomes. Notably, the control group had a higher percentage of perineal tears compared to the groups using lubricated massage, warm compresses, and hands-on techniques.

Sarhan et al. [3] conducted a quasi-experimental (comparative) study, dividing participants into three groups: a lubricant group, a warm compress group, and a control group. Their results indicated that warm compresses were effective in reducing perineal tears and limiting the extension of episiotomy. Specifically, the incidence of an intact perineum was significantly higher in the warm compress group (N = 30, 60%) compared to the control group (N = 5, 10%). The warm compress group also had a lower incidence of third- and fourth-degree perineal tears (N = 1, 2%) compared to the control group (N = 3, 6%). Additionally, the rate of episiotomy was notably lower in the warm compress group (N = 8, 16%) compared to the control group (N = 32, 64%).

El-Sayed and Lashin [13] found that 52% of women with perineal tears in the control group (N = 12) required perineal suturing. In contrast, only 21.7% (N = 5) of the warm compress group and 13.3% (N = 4) of the perineal massage group required suturing. Similarly, Fadlalmola et al. [14] concluded that the warm compress group showed superior results in terms of reducing episiotomy rates, the severity of perineal trauma, the need for perineal suturing, and pain scores.

Few studies have examined the effects of warm compresses on perineal trauma and pain in Saudi Arabia. Modoor et al. [9] conducted the only study in the central region, assessing the impact of warm compresses on perineal outcomes during the second stage of labor. This study supports the use of safe, natural techniques to reduce perineal tears, decrease the need for episiotomies, and alleviate perineal pain. The findings could aid hospitals in developing educational materials and strategies for pain management, as well as assist policymakers in formulating appropriate guidelines for using warm compresses during labor.

Objectives

The objectives of this study are to determine the effectiveness of warm compresses during the active phase of labor in reducing the incidence and severity of perineal tears, as well as the incidence of episiotomies. Additionally, the study aims to evaluate the impact of warm compresses on reducing perineal pain during labor.

## Materials and methods

Research purpose and design

This study aims to investigate the effect of warm compresses on the perineal area during the active phase of labor. It employs a quasi-experimental design, with one group serving as the control and the other as the intervention group. The research was conducted in the labor unit at East Jeddah Hospital.

Sample

In this study, a convenience sample of 80 mothers was divided into two groups: the intervention group (warm compression) and the control group (routine care). Participants were randomly assigned to each group after meeting the inclusion criteria and signing consent forms. Pain scores were recorded using the Visual Analogue Scale (VAS), as adopted by McCaffery and Beebe [15]. This self-reported scale is a 10 cm horizontal line ranging from 0 to 10, where 0 represents no pain and 10 represents extreme pain. Scores were categorized as no pain (0), mild pain (1-3), moderate pain (4-6), and severe pain (7-10).

Part III: perineal outcomes

The perineum status after delivery, including whether it was intact, involved an episiotomy, or had tears, was recorded. Additionally, if perineal tears were present, their degree was documented based on the physician's diagnosis and records.

Validation of tools and reliability

The study tool was developed based on relevant literature and reviewed for content validity by a jury of five obstetrics and gynecologic nursing specialists from King Abdulaziz University and Nursing College Faculty. The researcher refined the tool based on their feedback and suggestions, ensuring the accuracy of each question. The principal supervisor also reviewed the tool. The VAS, with its established validity, was used in its standardized form for the study.

Data collection process

The researcher interviewed each participant upon entry into the delivery room and collected the relevant data. After obtaining informed consent from all participants who met the inclusion criteria, the researcher divided the study sample into two groups: the perineal warm compresses group and the control group (without perineal warm compresses). Data collection was then conducted for both groups.

Intervention group (perineal warm compression)

The researcher applied the perineal warm compresses during active labor when the women were 6-7 cm dilated. The intervention group received warm compresses using a sterile abdominal towel soaked in water at a temperature ranging from 38°C to 44°C, as measured by a water thermometer (Brand SKY-TOUCH, waterproof (-50°C to 300°C)). The warm compress was applied between and during contractions for 15-20 minutes.

Control group

The control group received routine nursing care during active labor, which included monitoring and recording contractions, evaluating vital signs, monitoring the fetal heart, and assessing the frequency of micturition or catheterization to ensure bladder emptying. Additionally, a vaginal examination was performed every two hours.

Data analysis

The data were analyzed using IBM SPSS Statistics for Windows, Version 23.0 (Released 2015; IBM Corp., Armonk, NY, USA). Descriptive statistics included means, frequencies, percentages, and standard deviations. Inferential statistics involved chi-square and correlational tests, with results presented in tables and graphs. The accepted confidence interval was 95%, and a significance level of P < 0.05 was used for all analyses. Additionally, the study investigated the relationships between parity and perineal outcomes, as well as between newborn weight and perineal outcomes.

## Results

A total of 80 women participated in the study, with 40 in the control group and 40 in the intervention group. Table 1 shows the distribution of the two groups according to sociodemographic data. More than half of the intervention group (N = 27, 67.5%) and over three-quarters of the control group (N = 30, 75.5%) were aged between 28 and 38 years. In terms of education, nearly half of the intervention group had a high school education (N = 19, 47.5%), while almost half of the control group held a university degree (N = 19, 47.5%). Additionally, most participants in both groups were housewives (N = 35, 87.5% in the intervention group and N = 39, 92.5% in the control group).

**Table 1:** Sociodemographic data of the participants ^a^ Chi-square test

Variable	Total (N = 80)	Intervention group (N = 40)	Control group (N = 40)	Chi-square	P-value
Age^a^ (years)					
18 to <28	19 (23.8)	11 (27.5)	8 (20.0)	0.632	0.729
28 to <38	57 (71.2)	27 (67.5)	30 (75.5)		
38 and above	4 (5.0)	2 (5.0)	2 (5.0)		
Level of education^a^					
Illiterate	3 (3.8)	2 (5.0)	1 (2.5)	11.1	0.007
Read and write	17 (21.2)	4 (10.0)	13 (32.5)		
High school	26 (32.5)	19 (47.5)	7 (17.5)		
University and above	34 (42.5)	15 (37.5)	19 (47.5)		
Occupation^a^					
Housewife	72 (90.0)	35 (87.5)	37 (92.5)	0.722	0.835
Student	6 (7.5)	4 (10.0)	2 (5.0)		
Working	2 (2.5)	1 (2.5)	1 (2.5)		

Table 2 presents the distribution of the two groups (intervention and control) according to obstetrical history. The results show that nearly half of the intervention group (N = 19, 47.5%) and half of the control group (N = 20, 50.0%) had been pregnant four times or more. In terms of parity, 25.5% (N = 10) of the intervention group and 35% (N = 14) of the control group had delivered four times or more. Regarding abortion history, more than half of both groups did not have a history of abortion (intervention group: N = 29, 72.5%; control group: N = 27, 67.5%). No significant differences were observed in obstetrical history between the two groups.

**Table 2:** Obstetrical history of the participants ^a ^Chi-square test

Variable	Total (N = 80)	Intervention group (N = 40)	Control group (N = 40)	Chi-square	P-value
Gravidity^a^					
Primigravida	10 (12.5)	8 (20.0)	2 (5.0)	4.441	0.217
2	14 (17.5)	6 (15.0)	8 (20.0)		
3	17 (21.2)	7 (17.5)	10 (25.0)		
4+	39 (48.8)	19 (47.5)	20 (50.0)		
Parity^a^					
0	10 (12.5)	8 (20.0)	2 (5.0)	6.992	0.234
1	18 (22.5)	7 (17.5)	11 (27.5)		
2	18 (22.5)	9 (22.5)	9 (22.5)		
3	10 (12.5)	6 (15.0)	4 (10.0)		
4+	24 (30.0)	10 (25.5)	14 (35.0)		
Abortion^a^					
0	56 (70.0)	27 (67.5)	29 (72.5)	2.294	0.881
1	18 (22.5)	10 (25.0)	8 (20.0)		
2	4 (5.0)	2 (5.0)	2 (5.0)		
3	1 (1.2)	0 (0)	1 (2.5)		
4+	1 (1.2)	1 (2.5)	0 (0)		

Table 3 presents the distribution of the studied groups according to previous pregnancies. It shows that more than three-quarters of the intervention group (N = 31, 77.5%) and the majority of the control group (N = 37, 92.5%) were between 37 and 39 weeks of gestation. The perineum status after previous delivery indicated a tear in 32.5% of the intervention group (N = 13) and 40% of the control group (N = 16). Regarding perineal intactness, 35% of the intervention group (N = 14) and 22.5% of the control group (N = 9) had an intact perineum in their previous delivery. A significant difference was observed between the two groups in terms of previous perineum status (P < 0.05).

**Table 3:** History of previous pregnancies for the participants ^a^ Chi-square test and Fisher’s exact test

Variable	Total (N = 80)	Intervention group (N = 40)	Control group (N = 40)	Chi-square	P-value
Gestation of last delivery^a^					
Preterm	2 (2.5)	1 (2.5)	1 (2.5)	4.129	0.087
37-39 (term)	68 (85.5)	31 (77.5)	37 (92.5)		
Primigravida	10 (12.5)	8 (20.0)	2 (5.0)		
Perineum status^a^					
Intact	23 (28.8)	14 (35.0)	9 (22.5)	8.553	0.035
Episiotomy	18 (22.5)	5 (12.5)	13 (32.5)		
Tears	29 (36.2)	13 (32.5)	16 (40.0)		

Table 4 presents the distribution of both studied groups according to the initial assessments. The results indicate that about three-quarters of the intervention group (N = 28, 70.0%) and 82.5% of the control group (N = 33) were between 37 and 39 weeks of gestation. Regarding antenatal perineum massage during the previous three months, most participants did not use it (intervention group: N = 79, 97.5%; control group: N = 40, 100%). Similarly, the majority of participants in both groups had not performed Kegel exercises during the previous three months (intervention group: N = 39, 97.5%).

**Table 4:** Initial women’s assessment ^a ^Chi-square test

Variable	Total (N = 80)	Intervention group (N = 40)	Control group (N = 40)	Chi-square	P-value
Week of gestation^a^					
37-39	61 (76.2)	28 (70.0)	33 (82.5)	1.726	0.189
40-41	19 (23.8)	12 (30.0)	7 (17.5)		
Perineum massage^a^					
Yes	1 (1.2)	1 (2.5)	0 (0)	1.013	0.314
No	79 (98.8)	39 (97.5)	40 (100)		
Kegel exercise^a^					
Yes	2 (2.5)	1 (2.5)	1 (2.5)	1.2	0.753
No	78 (97.5)	39 (97.5)	39 (97.5)		
Duration of the first stage^a^					
<6	26 (32.5)	14 (35.0)	12 (30.0)	12.486	0.165
6 to <9	48 (61.3)	22 (55.0)	26 (65.0)		
9 to <12	6 (7.0)	4 (10)	2 (5.0)		
12 to <16	0 (0)	0 (0)	0 (0)		
Duration of the second stage^a^					
<1 hour	41 (51.3)	15 (37.5)	26 (65.0)	17.307	0.063
1-2	33 (41.3)	20(50.0)	13 (32.5)		
>2	6 (7.4)	5 (12.5)	1 (2.5)		
Pain relief^a^					
Yes	0 (0)	0 (0)	0 (0)	-	
No	80 (100)	40 (100)	40 (100)		
Epidural anesthesia^a^					
Yes	0 (0)	0 (0)	0 (0)	-	
No	80 (100)	40 (100)	40 (100)		

Regarding the duration of the first stage of labor, 65% (N = 26) of the control group and 55% (N = 22) of the intervention group reported a duration of six to nine hours. For the second stage of labor, 65.0% (N = 26) of the control group and 37.5% (N = 15) of the intervention group experienced a duration of less than one hour. None of the participants received pain relief or epidural anesthesia.

Table 5 presents the distribution of the studied groups according to pain scores for the second, third, and fourth stages of labor. The results indicate that the intervention and control groups had comparable pain scores, with the means of pain scores at all stages being higher in the control group. The researcher used mean and standard deviations to analyze these scores.

**Table 5:** Pain score for the second, third, and fourth stages of labor ^a^ t-test ^b ^Mann-Whitney U test

Variable	Intervention group (N = 40)	Control group (N = 40)
Second stage^a^	8.7 ± 1.7	9.7 ± 1.2
Third stage^a^	2.8 ± 2.7	4.5 ± 2.7
Fourth stage^b^	1.5 ± 2.2	2.9 ± 2.4

**Figure 1:**
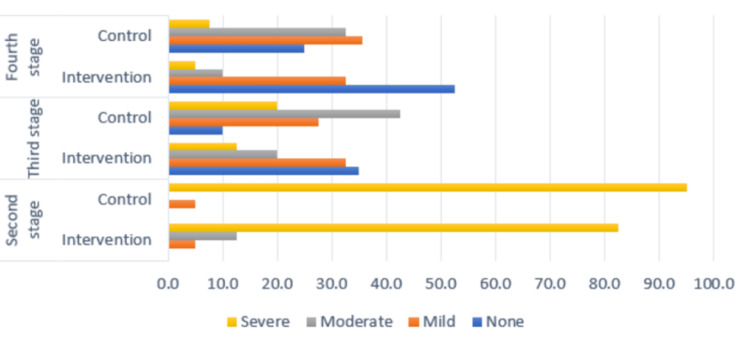
Pain levels by stage and studied group

Figure 1 illustrates the distribution of pain levels by stage and group. It shows that severe pain was more prevalent in the control group compared to the intervention group during the second stage of labor. Additionally, the intervention group reported a higher incidence of no pain during the third and fourth stages of labor compared to the control group.

Table 6 presents the distribution of perineal outcomes for the studied groups. The results show that less than half of the intervention group (N = 17, 42.5%) and nearly a third of the control group (N = 11, 27.5%) had intact perineal outcomes, indicating that the intervention group had a higher rate of intact perineum compared to the control group. However, less than half of the intervention group (N = 17, 42.5%) and more than half of the control group (N = 25, 62.5%) experienced perineal tears after delivery. Regarding episiotomy, 15.5% (N = 6) of the intervention group and 10% (N = 4) of the control group had tears. In terms of tear severity, more than one-third of the intervention group (N = 13, 32.5%) and half of the control group (N = 20, 50.0%) had first-degree tears, while 7.5% (N = 3) of the intervention group and 12.5% (N = 5) of the control group had second-degree tears. There was no significant difference between the two groups in perineal outcomes or the degree of tears.

**Table 6:** Perineal outcomes ^a ^Chi-square and Fisher’s exact test

Variable	All (N = 80)	Intervention (N = 40)	Control (N = 40)
Perineal assessment^a^			
Intact	28 (35.0)	17 (42.5)	11 (27.5)
Episiotomy	10 (12.5)	6 (15.5)	4 (10.0)
Tears	42 (52.5)	17 (42.5)	25 (62.5)
Degree of tears^a^			
First	33 (41.3)	13 (32.5)	20 (50.0)
Second	8 (10.0)	3 (7.5)	5 (12.5)
Third	1 (12.5)	1 (2.5)	0 (0)
Fourth	0 (0)	0 (0)	0 (0)
Intact or episiotomy	38 (47.5)	23 (57.5)	15 (37.5)

## Discussion

The study aims to investigate the effect of warm compresses on the perineal area during active labor and is among the first conducted in the western region of Saudi Arabia. The sociodemographic data of the sample are consistent with another Saudi study, which found that a large percentage of women were between the ages of 23 and 26, representing 48% of the population [9]. Similarly, in Egypt, the majority of women were aged between 25 and 30 [3], reflecting similar age patterns across different communities.

Regarding education, the study’s findings align with other Saudi research, where 55% of participants in both groups had a college education, with no significant differences observed [9]. Contrastingly, studies in Egypt showed that most participants had a university education, with no significant differences between groups [3]. In Iran, 65.4% of participants had a diploma-level education [16]. These variations in educational levels across studies may be attributed to regional differences and sample sizes.

In terms of obstetric history, the study’s findings diverged from those of an Egyptian study, which reported that most participants were primigravida [12]. In contrast, Modoor et al. [9] found that all participants in their Saudi study were primigravida. Another Egyptian study also focused exclusively on primigravida participants, differing from the current study’s sample [3]. These differences highlight the variability in study populations and the impact of regional and methodological factors on obstetric histories.

Pain intensity

The application of warm compresses significantly reduces labor pain during the first stage [17]. This study found that applying a warm compress to the perineum during active labor notably decreased perineal pain, with the mean pain score for the intervention group dropping more than that of the control group during the second, third, and fourth stages of labor. A similar effect was observed in a study by Farahmand et al. [11] in Iran, which investigated the impact of warm compresses on pain during birth. This study included 150 primigravid women divided into two groups; the intervention group experienced significantly less pain during the first stage of labor (mean score of 5.35) compared to the control group (mean score of 8.61), and during the second stage of labor, the pain score was 3.32 in the intervention group versus 7.40 in the control group. Significant differences in pain scores were noted between the two groups in the first and second stages (P < 0.05). The mean pain reduction observed in the current study was slightly less than that in the Iranian study, which may be attributed to differences in sample size.

In contrast to previous studies conducted in Saudi Arabia, which investigated the impact of warm compresses during the second stage of labor on perineal tear and pain with 50 primigravida women in each group, this study revealed that more than half (N = 26, 52.0%) of the intervention group reported moderate pain during the second stage of labor, while 46.0% (N = 23) of the control group reported mild pain [9]. Furthermore, an Iranian study involving 150 primigravida women found that three-quarters of the control group experienced severe pain during the second stage of labor (N = 51, 68%), compared to no pain reported in the intervention group [11]. This variation in outcomes can be attributed to differences in sample size and the duration of warm compress application to the perineal area. Similarly, the experimental study conducted by Gaheen and Abo-Hatab [12] demonstrated that women’s behavioral responses to perineal pain improved significantly with the use of warm compresses.

Perineal outcomes

According to the current research, the intervention group demonstrated better results compared to the control group in terms of intact perineal outcomes. In line with an experimental study conducted in Egypt, the results showed that 60% (N = 18) of the warm compress group and 16.7% (N = 5) of the control group achieved intact perineal outcomes [12]. Similarly, the study by Modoor et al. [9] revealed that 22% (N = 11) of the study group and 10% (N = 5) of the control group had intact perineal outcomes. The longer duration of perineal warm compress application in our study compared to Modoor et al.’s [9] study may account for the differences in results.

In our study, most participants in the control group experienced perineal tearing compared to the intervention group. This finding is consistent with Modoor et al. [9], who reported that 60% (N = 30) of the study group and 66% (N = 33) of the control group had perineal tear outcomes with statistical significance. Similar results were observed in a quasi-experimental study in Egypt, where the control group had more than double the incidence of perineal tears compared to the intervention group [3].

Regarding the degree of perineal tears, our study found a higher percentage of tears in the control group, with significant differences between the groups. This result aligns with Gaheen and Abo-Hatab [12], who found that first-degree tears were nearly five times more common in the control group than in the warm compress group. Conversely, Modoor et al. [9] reported that 61.3% (N = 19) of the study group and 31.7% (N = 13) of the control group had first-degree tears, which differs from our findings. El-Sayed and Lashin [13] conducted a randomized controlled clinical trial in Egypt to assess the effectiveness of massage versus warm compresses in preventing perineal injury. They found that first-degree tears affected the same percentage of participants in both the massage and warm compress groups.

Our results also align with those of Gaheen and Abo-Hatab [12], who found that warm compresses were effective in reducing the incidence of second-degree perineal tears, with 0% (N = 0) in the warm compress group compared to 30.8% (N = 4) in the control group. This finding is consistent with a randomized controlled trial in Malaysia by Goh et al. [2], which reported that combined massage and warm compresses reduced the incidence of significant second-degree perineal tears to 43% (N = 34) in the study group versus 66% (N = 51) in the control group. The study by Modoor et al. [9] also supports our results, with 32.3% (N = 10) of the study group and 58.5% (N = 24) of the control group experiencing second-degree tears.

Additionally, our study revealed that most primigravida participants in the control group underwent an episiotomy. This high incidence may be attributed to the common practice of performing episiotomies for primipara women. This contrasts with a study conducted in Iran by Alihosseni et al. [17], who investigated the impact of a perineal heating pad on the frequency of episiotomies and perineal tears. They found that warm compresses reduced the rate of episiotomies by 20.8% (N = 11) in the study group compared to 40.7% (N = 22) in the control group, with statistical significance. Magoga et al. [4] conducted a meta-analysis in Italy with 2,103 participants in randomized trials of warm compresses during the second stage of labor. This study did not align with our findings, as it reported that applying warm compresses during the second stage of labor reduces the risk of episiotomy. Additionally, combined massage and warm compresses were found to reduce the rate of episiotomy [4].

In summary, while perineal warm compresses did not significantly reduce the rate of episiotomy, they notably decreased the presence and severity of perineal tears. Furthermore, the study demonstrated that the intensity of perineal pain was reduced more effectively in the warm compress group compared to the control group.

Limitations and recommendations

The limitations of this study include the small sample size due to time constraints on data collection and the fact that the data were collected from only one hospital, which may limit the generalizability of the findings. Additionally, the practice of performing unnecessary episiotomies on primigravida women by some physicians may have influenced the outcomes.

Based on the study results, applying warm compresses to the perineum appears to effectively minimize perineal tears and reduce pain intensity. Future research should explore the effects of perineal warm compresses during both the latent and active phases of labor and investigate their combined use with other techniques, such as perineal massage.

## Conclusions

The current study’s findings indicate that applying a warm compress to the perineal area during the active phase of labor can enhance perineal intactness. The study demonstrated that this method effectively reduces perineal pain, with the intervention group experiencing significantly less pain during the third and fourth stages of labor compared to the control group. Additionally, using a warm compress is a nonpharmacological, cost-effective, easy-to-administer, and safe approach that nursing staff can perform without requiring additional training. However, the study found no significant impact on the incidence of episiotomies.

## Figures and Tables

**Figure 2:**
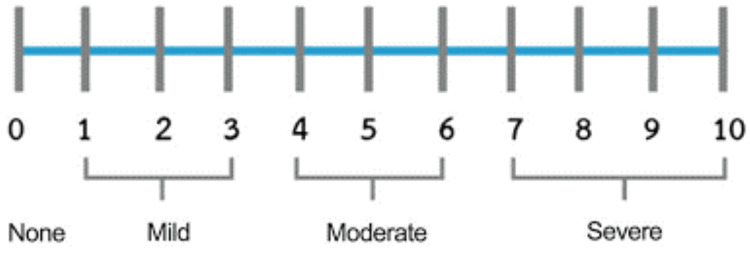
Pain score of the second stage of labor

**Figure 3:**
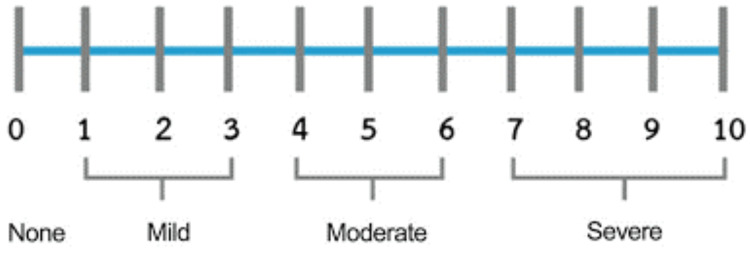
Pain score of the third stage of labor (before the placenta delivered)

**Figure 4:**
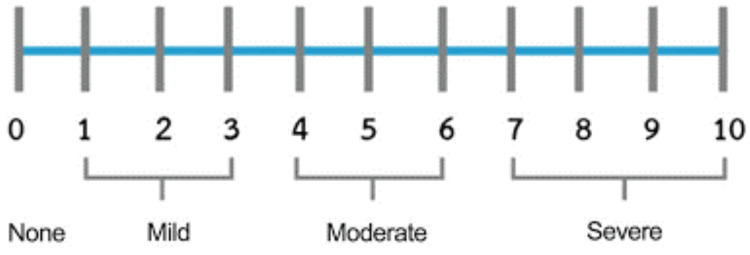
Pain score of the fourth stage of labor (before breastfeeding and after suturing)

## Appendices

Part (1): Sociodemographic data

Section (1): Demographic data

1. Participant ID

2. Age (years)

o   18 to <28

o   28 to <39

o   ≥39

3. Level of education

o   Illiterate

o   Read and write

o   High school education

o   University education and higher

4. Occupation

Working

Housewife

Student

Section (2): Obstetrical history

1. Gravidity

o   Primigravida

o   1

o   2

o   3

o    ≥4

2. Parity

o   Primigravida

o   1

o   2

o   3

o   ≥ 4

3. Abortion

o   0

o   1

o   2

o   3

o   ≥4

History of previous pregnancy if multipara will be completed only

1. Gestation of last delivery( weeks)

o   <37 (preterm)

o   37-39 (term)

o   40-41 (postdate)

2. Mood of last delivery

o   Spontaneous vaginal delivery

o   Cesarean delivery

o   Assistance delivery

3. Status of the perineum area of Last delivery

o   Intact

o   Episiotomy

o   Tears

Section (3) Initial women assessment

1. Week of gestation

o   37-39

o   40-41

2. Applying an antenatal perineum massage

o   Yes

o   No

3. Applying Kegel exercise during the last three months

o   Yes

o   NO

4. Current history of labor

Duration of the first stage (hours):

o   <6

o   6 to <9

o   9 to <12

o   12 to <1

Duration of the second stage

o   <1

o   1-2

o   >2

Received pain relief

o   Yes

o   No

Epidural anesthesia

o   Done

o   Not fone

Part (2) Perineal outcomes

1.  Perineal outcomes assessment

o   Intact

o   Episiotomy

o   Tears

2. Describing the degree of perineal tear if there is a tear

o   First degree

o   Second degree

o   Third degree

o   Fourth degree

Part (3): Perinium Pain (VAS)
